# Anti-fatigue Performance in SSVEP-Based Visual Acuity Assessment: A Comparison of Six Stimulus Paradigms

**DOI:** 10.3389/fnhum.2020.00301

**Published:** 2020-07-31

**Authors:** Xiaowei Zheng, Guanghua Xu, Yubin Zhang, Renghao Liang, Kai Zhang, Yuhui Du, Jun Xie, Sicong Zhang

**Affiliations:** ^1^School of Mechanical Engineering, Xi’an Jiaotong University, Xi’an, China; ^2^State Key Laboratory for Manufacturing Systems Engineering, Xi’an Jiaotong University, Xi’an, China

**Keywords:** steady-state visual evoked potential, visual acuity, mental fatigue, anti-fatigue performance, stimulus paradigm

## Abstract

**Purpose:**

The occurrence of mental fatigue when users stare at stimuli is a critical problem in the implementation of steady-state visual evoked potential (SSVEP)-based visual acuity assessment, which may weaken the SSVEP amplitude and signal-to-noise ratio (SNR) and subsequently affect the results of visual acuity assessment. This study aimed to explore the anti-fatigue performance of six stimulus paradigms (reverse vertical sinusoidal gratings, reverse horizontal sinusoidal gratings, reverse vertical square-wave gratings, brief-onset vertical sinusoidal gratings, reversal checkerboards, and oscillating expansion–contraction concentric rings) in SSVEP acuity assessment.

**Methods:**

Based on four indices of α + θ index, pupil diameter, National Aeronautics and Space Administration Task Load Index (NASA-TLX), and amplitude and SNR of SSVEPs, this study quantitatively evaluated mental fatigue in six SSVEP visual attention runs corresponding to six paradigms with 12 subjects.

**Results:**

These indices of mental fatigue showed a good agreement. The results showed that the paradigm of motion expansion–contraction concentric rings had a superior anti-fatigue efficacy than the other five paradigms of conventional onset mode or pattern reversal mode during prolonged SSVEP experiment. The paradigm of brief-onset mode showed the lowest anti-fatigue efficacy, and the other paradigms of pattern reversal SSVEP paradigms showed a similar anti-fatigue efficacy, which was between motion expansion–contraction mode and onset mode.

**Conclusion:**

This study recommended the paradigm of oscillating expansion–contraction concentric rings as the stimulation paradigm in SSVEP visual acuity because of its superior anti-fatigue efficacy.

## Introduction

Recently, there have been some research findings across a range of applications in vision science based on steady-state visual evoked potential (SSVEP) (Norcia et al., 2015; Odom et al., 2016; Zheng et al., 2019b). As an essential part of any ophthalmological or optometric examination, visual acuity is the most commonly measured visual function (Fahad et al., 2008). Within 40 years, the SSVEP technique has been used for measuring visual acuity in some studies, demonstrating that SSVEP provides an objective and quantitative method in visual acuity assessment, especially for infants or individuals with intellectual disabilities, hysteria, or malingering (Tyler et al., 1979; Norcia and Tyler, 1985a, b; Hemptinne et al., 2018).

There are some parameters, such as electrode placement, temporal frequency, stimulus area, and sweep duration, related to SSVEP visual acuity assessment, and some studies have given their recommended parameter settings (Yadav et al., 2009; Almoqbel et al., 2011; Hemptinne et al., 2018). As for stimulus paradigms used in SSVEP visual acuity assessment, previous studies have compared some performance, such as sensitive electrodes, harmonic components of SSVEP response, correlation, and agreement between objective SSVEP and subjective psychophysical visual acuity, of six paradigms (reverse vertical sinusoidal gratings, reverse horizontal sinusoidal gratings, reverse vertical square-wave gratings, brief-onset vertical sinusoidal gratings, reversal checkerboards, and oscillating expansion–contraction concentric rings) (Tobimatsu et al., 1993; Chen et al., 2019; Zheng et al., 2019a, 2020; Hamilton et al., 2020).

However, although SSVEP can be an objective method to assess visual acuity, mental fatigue caused by uncomfortable light twinkling and contrast changes of prolonged visual stimulus can decrease arousal level and attention, worsening the SSVEP signal quality and consequently degrading the practical performance (Lee et al., 2010; Zhu et al., 2010; Cao et al., 2014; Chen et al., 2015). Previous studies have indicated that the amplitude and the signal-to-noise ratio (SNR) are related to the mental fatigue of the subjects, with decreasing amplitude and SNR corresponding to the developing fatigue (Wu et al., 2010), which can affect the precision and the accuracy of SSVEP visual acuity results since the threshold determination criterion of SSVEP visual acuity is related to the amplitude and the SNR of electroencephalography (EEG) response (Fahad et al., 2008; Yadav et al., 2009).

To evaluate the mental fatigue of prolonged SSVEP task, previous studies have proved that EEGs in the α band (8–13 Hz) and the θ band (4–7 Hz) can be adapted to assess mental fatigue (Klimesch, 1999; Cao et al., 2014; Kathner et al., 2014; Xie et al., 2016). The θ activity is related to drowsiness, while the α waves appear during relaxed conditions, at decreased attention levels and in a drowsy but wakeful state (Klimesch, 1999). Increased fatigue level is often related to the global increase of EEG power in the α and the θ bands (Klimesch, 1999; Xie et al., 2016). Moreover, pupil diameter can also be an index to evaluate mental fatigue, and the increase of mental fatigue coincides with a decrease in pupil diameter (Hopstaken et al., 2015b; Koo et al., 2018). Besides that, the National Aeronautics and Space Administration Task Load Index (NASA-TLX) is also used as a subjective and quantitative estimation of mental fatigue (Hart and Staveland, 1988; Sampei et al., 2016).

On this basis, in this study, four indices, i.e., the EEG spectral powers of α + θ, SSVEP properties of amplitude and SNR, pupil diameters recorded by the eye tracker, and subjective NASA-TLX, were measured in six SSVEP visual attention runs corresponding to six previously mentioned types of paradigms to compare their anti-fatigue performance (Cao et al., 2014; Xie et al., 2016). We hypothesized that the reversal vertical sinusoidal gratings, reverse horizontal sinusoidal gratings, reverse vertical square-wave gratings, and reversal checkerboards would show a similar anti-fatigue performance since the stimulus mode and the pattern were similar. When staring at the brief-onset vertical sinusoidal gratings, the subjects would become more fatigued because of the constantly changing brightness of the onset and offset mode. As for the oscillating expansion–contraction concentric rings, since the overall brightness was uniform when evoking steady-state motion visual evoked potential (SSMVEP) (Xie et al., 2016; Zheng et al., 2019a), its anti-fatigue property would be better than that of other stimulus paradigms.

## Materials and Methods

### Subjects

Twelve subjects (two females), aged between 21 and 25 years old and with normal or corrected normal visual acuity, participated in this experiment. They had no history of eye disease. All the subjects gave informed written consent following a protocol approved by the institutional review board of Xi’an Jiaotong University, conforming to the Declaration of Helsinki.

### EEG Recordings

In this study, EEG signals were recorded from six occipital electrodes (PO3, PO4, POz, O1, O2, and Oz) according to the 10–20 system with a ground electrode, Fpz, placed on the forehead and a reference electrode, A1, placed on the left earlobe (Listed, 2006). The EEG signals were collected by a g.USBamp acquisition and processing system and an active electrode system g.GAMMAbox (g.tec, Schiedlberg, Austria) at a sampling rate of 1,200 Hz. Besides that, an online band-pass filter from 2 to 100 Hz was imposed to remove artifacts, and an offline notch filter between 48 and 52 Hz was applied to eliminate the power line interference.

### Stimulus Designs

**FIGURE 1:**
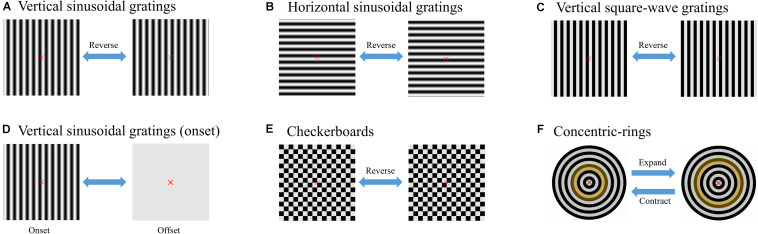
Examples of six stimulus paradigms (Zheng et al., 2020). **(A)** Reverse vertical sinusoidal gratings. **(B)** Reverse horizontal sinusoidal gratings. **(C)** Reverse vertical square-wave gratings. **(D)** Brief-onset vertical sinusoidal gratings. **(E)** Reversal checkerboards. **(F)** Oscillating expansion–contraction concentric rings. The yellow ring indicates the shape shifting of the same zone during different processes in the paradigm of concentric rings.

As shown in Figure 1, six stimulus paradigms (A: reverse vertical sinusoidal gratings, B: reverse horizontal sinusoidal gratings, C: reverse vertical square-wave gratings, D: brief-onset vertical sinusoidal gratings, E: reversal checkerboards, and F: oscillating expansion–contraction concentric rings) were introduced as six separate experimental runs (Zheng et al., 2020). As for each run, one stimulator was presented to the subjects at the center of a 24.5-in. LCD monitor (PG258Q, ASUS, Taipei, China) with a resolution of 1,920 × 1,080 pixels and a refresh rate of 240 Hz.

The subjects were asked to sit 60 cm away from the monitor with the center at eye level. The visual angle of the stimulator was 4° with a diameter of 148 pixels, in accordance with the recommended visual angle parameter of previous studies (Almoqbel et al., 2011; Ng et al., 2012). The reversal or oscillating details of the six paradigms were the same as in our previous studies, with a contrast of 99.7%, and the duty cycle of paradigm D remained at 0.3 (Zheng et al., 2019a, 2020). According to previous studies (Almoqbel et al., 2011), the spatial frequency of three cycles per degree (cpd) corresponding to 1.0 logMAR optotype and temporal frequency of 7.5 Hz was assigned to all six stimulus paradigms. In the whole experiment, a spatially homogeneous white background with luminance of 208 cd/m^2^ was displayed in pauses and around the stimulators. The stimulus paradigms were controlled by MATLAB (MathWorks, Natick, United States) with the Psychophysics Toolbox (Brainard, 1997).

### Experimental Procedure

For each subject, six runs A, B, C, D, E, and F corresponding to six stimulus paradigms A, B, C, D, E, and F were carried out, respectively. An eye tracker (Tobii X2-30, Stockholm, Sweden) was used to monitor the subjects’ eye movements and record their pupil diameter at a sample rate of 30 Hz. Each run consisted of 23 trials with three pre-experimental trials and 20 experimental trials. Each trial lasted 5 s, with an interval of 0.5 s between two trials. During the first three pre-experimental trials, to measure the baseline mental fatigue level from baseline pupil diameter and α + θ band, the subjects stared at a black screen with only a red fixation cross at the position of the center stimulator, so there was no interference from the pupillary light reflexes of the eye to the environmental lighting (Hopstaken et al., 2015a). As for the other 20 trials, the stimulator was presented and the subjects were instructed to binocularly maintain attention on the center target stimulus throughout the experiment. The order of the six runs was random, and there was enough rest time for the subjects between two runs as long as the subjects wished. Additionally, a red fixation cross was presented at the center of the paradigms to aid fixation (Almoqbel et al., 2011). The whole experiment of each subject usually lasted for about 30–45 min, depending on the inter-run rest time governed by the subjects.

### NASA-TLX

NASA-TLX, originally developed as a paper-and-pencil questionnaire by NASA Ames Research Center’s Sandra Hart in the 1980s, has become a gold standard for measuring subjective workload across a wide range of applications (Hart and Staveland, 1988; Hart, 2006). Here we used NASA-TLX to assess mental fatigue subjectively as a psychological measurement. We assumed that workload deduced by the NASA-TLX represented the mental fatigue of the subjects (Sampei et al., 2016).

Firstly, the six defined sources of workload – mental demand (MD), physical demand (PD), temporal demand (TD), performance (PE), effort (EF), and frustration (FR) – were explained to the subjects.

The instruction was in Chinese as it is their native language. Then, after the subjects completed each run, they were asked to evaluate the six factors on a 0–100 scale. Next, after all the runs were completed, the subjects were asked to complete a pairwise comparison method of the six defined sources. The weights *a*, *b*, *c*, *d*, *e*, and *f* were assigned to each of the six workload sources from the pairwise comparison results, with weight integers ranging from 0 to 5, and their combinations were *C*(6, 2) = 15 (Sampei et al., 2016). Finally, the individual NASA-TLX of each run for each subject was derived from a weighted average of the ratings of these six factors:

(1) $$\begin{array}{c} \text{NASA}-\text{TLX}\\ =\frac{a*\text{MD}+b*\text{PD}+c*\text{TD}+d*\text{PE}+e*\text{EF}+f*\text{FR}}{15}. \end{array}$$

### Signal Processing of the EEG Data

#### Canonical Correlation Analysis

Canonical correlation analysis (CCA) is a non-parametric multivariable method used to reflect the overall linear correlation between two groups of variables, and it is also used in the analysis of SSVEPs (Lin et al., 2007; Bin et al., 2009; Yan et al., 2018). In our study, it was also used to describe the correlations between the multi-channel SSVEP signals *X* and reference signal *Y*_*i*_. *X* is the six-electrode channel signal in each trial. The reference signal *Y*_*i*_ composed of sine and cosine pairs is constructed at the reference frequency *f*_*i*_ (*i* = 1, 2, …, *N*):

(2) $$Y_{i}=\left(\begin{array}{c} \sin\left(2\pi f_{i}t\right)\\ \cos\left(2\pi f_{i}t\right) \end{array}\right),t=\frac{1}{F_{\text{s}}},\ldots ,\frac{S}{F_{\text{s}}}.$$

where *F*_s_ is the sampling rate, and *S* is the sample point. Here the reference frequency *f*_*i*_ is set to 1.0, 1.1, …, 35.0 Hz (i.e., *N* = 341).

The linear transformations of *X* and *Y*_*i*_ are *x* = *w_*x*_^*T*^X* and *y_*i*_* = *w_*yi*_^*T*^Y_*i*_*, respectively, and the maximum correlation coefficient value ρ*_*i*_* between *X* and *Y*_*i*_ can be calculated by the CCA method as:

(3) $$\rho_{i}=\max_{w_{x},w_{y}}\frac{E\left[w_{x}^{T}XY_{i}^{T}w_{yi}\right]}{\sqrt{E\left[w_{x}^{T}XX^{T}w_{x}\right]E\left[w_{yi}^{T}Y_{i}Y_{i}^{T}w_{yi}\right]}}.$$

where *E* denotes the symbol of the expected value in statistics, and the superscript *T* indicates the transposed matrix. The maximum correlation coefficient value ρ*_*i*_*, which represents the maximum correlation between *X* and *Y*_*i*_, can be considered as the response to the stimulus paradigm of SSVEPs at the reference frequency *f*_*i*_ (*f*_1_, *f*_2_…, *f*_*N*_). Therefore, all the ρ*_*i*_* and their corresponding frequency *f*_*i*_ can be plotted as a CCA spectrum. The ρ*_*i*_* at the stimulus frequency of 7.5 Hz was regarded as the SSVEP amplitude.

#### Signal-to-Noise Ratio

Signal-to-noise ratio (SNR) refers to the ratio of signal to noise in a device or system. In our study, the SNR was defined as the ratio of the square of the CCA coefficient at the stimulus frequency of 7.5 Hz to the mean value of the square of the *n* adjacent points on the CCA spectrum:

(4) $$\text{SNR}=\frac{z{\left(f\right)}^{2}}{\frac{1}{n}*\sum_{k=1}^{\frac{n}{2}}\left[z{\left(f+c*k\right)}^{2}+z{\left(f-c*k\right)}^{2}\right]}.$$

where *n* is set to 10, and *f* is 7.5 Hz. *z*(*f*) is the CCA coefficient of the stimulus frequency *f* on the CCA spectrum. Then, *c* is the scale value of abscissa on the CCA spectrum, which is set to 0.1.

#### EEG Spectral Powers of α + θ Band

Common average reference (CAR) fusion is a commonly used EEG spatial filtering method performed by subtracting the mean of all electrode signals from the selected electrode signals to enhance the SNR of the selected electrode signals (Friman et al., 2007; Yan et al., 2019). In this study, we chose Oz electrode in spectral analysis, so the time domain EEG signal *V*_*i*_ to be analyzed can be expressed as:

(5) $$V_{i}=V_{Oz}-\frac{1}{6}\sum_{j=1}^{6}V_{j},$$

where *V*_*j*_ is the EEG signal from six electrode channels (PO3, PO4, POz, O1, O2, and Oz).

As for the processing of the EEG signals, firstly, a band-pass filter of 3–45 Hz was carried out to remove low-frequency drift and high-frequency interference. Then, CAR fusion was used for spatial filtering in each trial. Next, the Welch power spectrum density (PSD) in bins of 0.1 Hz was used for spectral analysis to obtain the EEG spectral powers of the α band of 8–13 Hz and the θ band of 4–7 Hz. Finally, the sum value of the PSD amplitude in the frequency band on the Welch power spectrum was defined as the EEG band power indices of the frequency bands of α and θ (Cao et al., 2014). Hence, the EEG combined index (α + θ) in each trial was obtained.

As the stimulation time and the experimental trial increased, the subject could get more fatigued. Hence, the mean values and SD of each index in the 1–5, 6–10, 11–15, and 16–20 experimental trials of each run were used to represent the corresponding four fatigue levels (i.e., level 1, level 2, level 3, and level 4), respectively (Xie et al., 2016). Fatigue level 4 represented the most fatigued state, while level 1 represented the least fatigued state.

### Statistical Analysis

Statistical analyses were carried out using SPSS 22.0 (IBM, Armonk, United States). One-way or two-way repeated-measures analysis of variance (ANOVA) with a significance of *P* < 0.05 was employed to evaluate the significance of changes in the four indices of α + θ index, SSVEP amplitude and SNR, pupil diameter index, and NASA-TLX index of six paradigms at two fatigue levels, i.e., fatigue level 1 and level 4. The *post-hoc* analysis with Bonferroni correction method for multiple comparisons was also used when necessary. Besides that, we used equal signs and inequality signs to visualize the anti-fatigue performance among the six paradigms based on each index.

## Results

### Pre-experimental Trials

**FIGURE 2:**
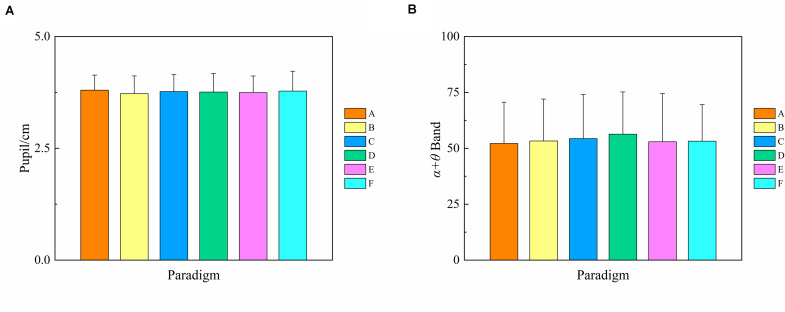
Comparison of the mean values and SD of the pupil diameter and α + θ band index of the first three pre-experimental trials for each paradigm over 12 subjects. Statistics were assessed by one-way repeated-measures ANOVA. **(A)** Pupil diameter index. **(B)** α + θ band index.

As for each subject, we assumed that the initial mental fatigue was the same at the beginning of each run since there was enough rest time between two runs and the order of the presentation of the six runs was random. Here, to verify this assumption, we estimated the difference of initial baseline mental fatigue among six runs corresponding to six paradigms. As the first three pre-experimental trials of each run were presented with a black background, the mean fatigue level of the first three pre-experimental trials can be regarded as the initial mental fatigue for each run. One-way repeated-measures ANOVA was used to analyze the difference in pupil diameter and α + θ band of the first three pre-experimental trials for each paradigm. As shown in Figure 2, there was no significant difference both in the pupil diameter and the α + θ band of the first three pre-experimental trials for each paradigm [*F*_(__5,55__)_ = 0.687, *P* = 0.635 for pupil diameter; Greenhouse–Geisser correction: *F*_(__3.238,35.623__)_ = 0.774, *P* = 0.525 for the α + θ band], demonstrating that our assumption that the initial mental fatigue was the same at the beginning of each run was credible.

### Comparison of NASA-TLX

**FIGURE 3:**
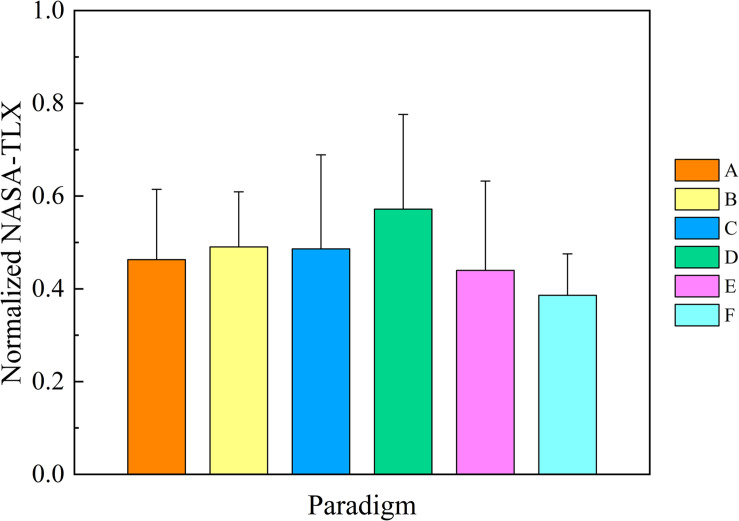
Comparison of the mean values and SD of normalized National Aeronautics and Space Administration Task Load Index for six stimulus paradigms over 12 subjects. Statistics were assessed by one-way repeated-measures ANOVA.

For the convenience of data analysis, the NASA-TLX of psychological measurement of mental fatigue was normalized per subject by his/her maximal value of six workload sources. The mean values and SD of normalized NASA-TLX for six stimulation paradigms over 12 subjects are shown in Figure 3. One-way repeated-measures ANOVA revealed that there was a significant difference in NASA-TLX among six paradigms [*F*_(__5,55__)_ = 0.074, *P* = 0.044]. As the corresponding Bonferroni *post-hoc* analysis shows in Table 1, there was no difference in the mean values of NASA-TLX among paradigms A, B, C, and E, demonstrating that these pattern reversal paradigms had a similar stimulus intensity for the human eyes. Paradigm D had the highest mean value of NASA-TLX than the other paradigms (*P* < 0.05, respectively), demonstrating that visual stimulation of onset mode had a high stimulus intensity, which may be due to the repetitive attentional demands of continuous flicker and contrast change (Xie et al., 2017). Except for paradigms C and E with a slightly but non-significantly higher NASA-TLX than paradigm F, there was a significant difference in the mean values of NASA-TLX between paradigms F and A, B, and D (*P* < 0.05, respectively), showing that paradigm F had the lowest value of NASA-TLX, in favor of that motion expansion–contraction SSMVEP stimulation which exhibited a superior anti-fatigue efficacy over the conventional flickering or pattern reversal SSVEP stimulation (Xie et al., 2016). Hence, the anti-fatigue performance of six paradigms based on NASA-TLX was as follows: F > E = A = B = C > D.

**TABLE 1:** Bonferroni *post-hoc* analysis of National Aeronautics and Space Administration Task Load Index among six stimulus paradigms.

Paradigm	B	C	D	E	F
A	*P* = 0.273	*P* = 0.436	*P* = 0.010*	*P* = 0.374	*P* = 0.038*
B	–	*P* = 0.828	*P* = 0.016*	*P* = 0.183	*P* < 0.001***
C	–	–	*P* = 0.008**	*P* = 0.058	*P* = 0.054
D	–	–	–	*P* = 0.005**	*P* = 0.002**
E	–	–	–	–	*P* = 0.302
F	–	–	–	–	–

***P < 0.001; **P < 0.01; *P < 0.05.

### Comparison of SSVEP Amplitude and SNR

**FIGURE 4:**
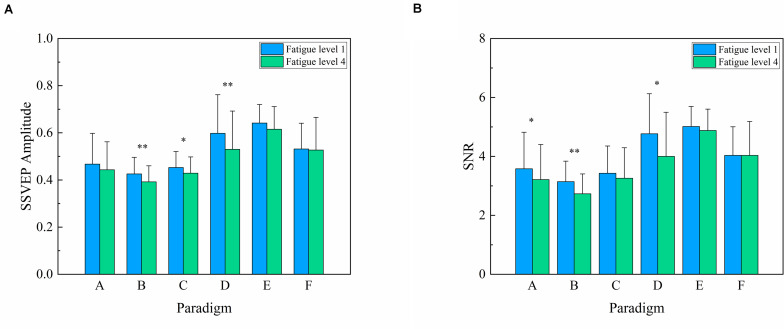
Comparison of the mean values and SD of steady-state visual evoked potential amplitude and signal-to-noise ratio (SNR) between fatigue level 1 and fatigue level 4 for each paradigm over 12 subjects. **(A)** Amplitude. **(B)** SNR. Statistics were assessed by one-way repeated-measures ANOVA. ^∗∗^*P* < 0.01; ^∗^*P* < 0.05.

To compare the changes in amplitude and SNR, the mean values and SD of the SSVEP amplitude and SNR summed over the stimulus frequency of 7.5 Hz in the 1–5 and 16–20 trials of each run were grouped to represent fatigue levels 1 and 4, respectively, as shown in Figure 4. Two-way repeated-measures ANOVA revealed that the interaction of two factors of “stimulus paradigm” and “fatigue level” yielded significance in SSVEP amplitude [*F*_(__5,55__)_ = 2.955, *P* = 0.020] and SNR [*F*_(__5,55__)_ = 2.695, *P* = 0.030]. Subsequently, one-way repeated-measures ANOVA found a significant difference in SSVEP amplitude among six paradigms at fatigue level 1 [Greenhouse–Geisser *F*_(__2.510,27.609__)_ = 14.116, *P* < 0.001] and fatigue level 4 [Greenhouse–Geisser *F*_(__2.637,29.006__)_ = 11.019, *P* < 0.001]. As the corresponding Bonferroni *post-hoc* analysis of SSVEP amplitude among six paradigms at fatigue level 1 and level 4 shows in Tables 2, 3, the SSVEP amplitude induced by different stimulus paradigms was different. Paradigms E and D induced the highest amplitude, and paradigms A, B, and C induced the lowest amplitude, with paradigm F in between.

**TABLE 2:** Bonferroni *post-hoc* analysis of steady-state visual evoked potential amplitude among six paradigms at fatigue level 1.

Paradigm	B	C	D	E	F
A	*P* = 1.000	*P* = 1.000	*P* = 0.228	*P* = 0.204	*P* = 1.000
B	–	*P* = 1.000	*P* = 0.022*	*P* < 0.001***	*P* = 0.006**
C	–	–	*P* = 0.116	*P* < 0.001***	*P* = 0.086
D	–	–	–	*P* = 1.000	*P* = 1.000
E	–	–	–	–	*P* = 0.017*
F	–	–	–	–	–

***P < 0.001; **P < 0.01; *P < 0.05.

**TABLE 3:** Bonferroni *post-hoc* analysis of steady-state visual evoked potential amplitude among six paradigms at fatigue level 4.

Paradigm	B	C	D	E	F
A	*P* = 0.695	*P* = 1.000	*P* = 1.000	*P* = 0.018*	*P* = 1.000
B	–	*P* = 0.343	*P* = 0.109	*P* < 0.001***	*P* = 0.024*
C	–	–	*P* = 0.459	*P* < 0.001***	*P* = 0.094
D	–	–	–	*P* = 1.000	*P* = 1.000
E	–	–	–	–	*P* = 0.533
F	–	–	–	–	–

***P < 0.001; **P < 0.01; *P < 0.05.

One-way repeated-measures ANOVA was also used to analyze the difference in SSVEP amplitude for paradigms A, B, C, D, E, and F between fatigue level 1 and level 4 and found a significant decrease in paradigms B, C, and D [*F*_(__1,11__)_ = 12.201, *P* = 0.005 for paradigm B; *F*_(__1,11__)_ = 5.047, *P* = 0.046 for paradigm C; *F*_(__1,11__)_ = 13.749, *P* = 0.003 for paradigm D]. The same trend of decrease was also found in paradigms A and E without statistical significance [*F*_(__1,11__)_ = 2.550, *P* = 0.139 for paradigm A; *F*_(__1,11__)_ = 4.353, *P* = 0.061 for paradigm E]. However, there was no obvious change in amplitude for paradigm F [*F*_(__1,11__)_ = 0.083, *P* = 0.779].

A similar significant difference of SNR results can also be found among six paradigms at fatigue level 1 [Greenhouse–Geisser *F*_(__2.427,26.696__)_ = 11.949, *P* < 0.001] and fatigue level 4 [Greenhouse–Geisser *F*_(__2.688,29.569__)_ = 10.594, *P* < 0.001] by one-way repeated-measures ANOVA. The corresponding Bonferroni *post-hoc* analysis of SSVEP SNR among six paradigms at fatigue level 1 and level 4 is shown in Tables 4, 5, revealing that paradigms E and D had the highest SNR and paradigms A, B, and C had the lowest SNR, with paradigm F in between.

**TABLE 4:** Bonferroni *post-hoc* analysis of steady-state visual evoked potential signal-to-noise ratio among six paradigms at fatigue level 1.

Paradigm	B	C	D	E	F
A	*P* = 1.000	*P* = 1.000	*P* = 0.311	*P* = 0.025	*P* = 1.000
B	–	*P* = 0.654	*P* = 0.043*	*P* < 0.001***	*P* = 0.060
C	–	–	*P* = 0.198	*P* < 0.001***	*P* = 0.298
D	–	–	–	*P* = 1.000	*P* = 0.501
E	–	–	–	–	*P* = 0.015*
F	–	–	–	–	–

***P < 0.001; **P < 0.01; *P < 0.05.

**TABLE 5:** Bonferroni *post-hoc* analysis of steady-state visual evoked potential signal-to-noise ratio among six paradigms at fatigue level 4.

Paradigm	B	C	D	E	F
A	*P* = 1.000	*P* = 1.000	*P* = 1.000	*P* = 0.008	*P* = 0.734
B	–	*P* = 0.576	*P* = 0.274	*P* < 0.001***	*P* = 0.044*
C	–	–	*P* = 1.000	*P* < 0.001***	*P* = 0.247
D	–	–	–	*P* = 1.000	*P* = 1.000
E	–	–	–	–	*P* = 0.134
F	–	–	–	–	–

***P < 0.001; **P < 0.01; *P < 0.05.

One-way repeated-measures ANOVA found that SNR had a similar decrease for paradigms A, B, and D between fatigue level 1 and level 4 [*F*_(__1,11__)_ = 6.175, *P* = 0.030 for paradigm A; *F*_(__1,11__)_ = 12.471, *P* = 0.005 for paradigm B; *F*_(__1,11__)_ = 8.584, *P* = 0.014 for paradigm D]. The same but non-significant trend of decrease was also found in paradigm C [*F*_(__1,11__)_ = 1.103, *P* = 0.316] and paradigm E [*F*_(__1,11__)_ = 1.341, *P* = 0.271]. However, there was no obvious change in SNR for paradigm F [*F*_(__1,11__)_ < 0.001, *P* = 0.999].

This implied that the factor of the types of stimulus paradigm had a significant influence on the SSVEP response during prolonged usage. Both SSVEP amplitude and SNR between fatigue level 1 and fatigue level 4 had a downtrend for paradigms A, B, C, D, and E, but paradigm F did not present a significant change in SSVEP amplitude and SNR between fatigue level 1 and level 4. These results were also in line with previous studies such that paradigm F of motion SSMVEP stimulation exhibited a superior anti-fatigue efficacy than conventional flickering or pattern reversal SSVEP stimulation during prolonged SSVEP visual acuity assessment (Xie et al., 2016). Hence, the anti-fatigue performance of six paradigms based on SSVEP amplitude and SNR was as follows: F > E > A = C = B ≥ D.

### Comparison of Pupil Diameter Index

**FIGURE 5:**
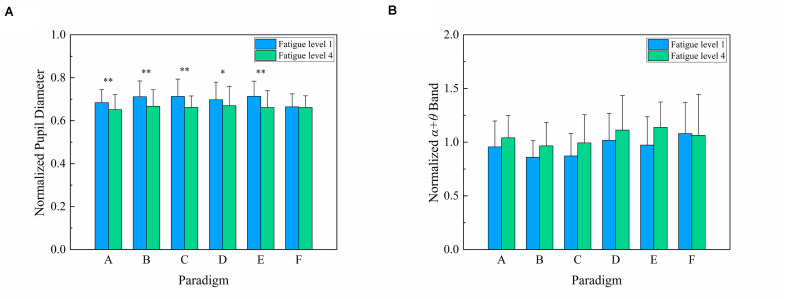
Comparison of the mean values and SD of normalized pupil diameter index and normalized α + θ index between fatigue level 1 and fatigue level 4 for six stimulus paradigms over 12 subjects. **(A)** Normalized pupil diameter index. Statistics were assessed by repeated-measures ANOVA. ^∗∗^*P* < 0.01; ^∗^*P* < 0.05. **(B)** Normalized α + θ index.

For the convenience of data analysis, the pupil diameter corresponding to each paradigm was normalized for each subject by his/her respective baseline pupil diameter of the first three pre-experimental trials of each paradigm. Figure 5A presents the normalized pupil diameter index between fatigue level 1 and fatigue level 4 for six stimulus paradigms over 12 subjects. Two-way repeated-measures ANOVA revealed that the interaction of two factors of “stimulus paradigm” and “fatigue level” yielded significance in normalized pupil diameter index [*F*_(__5,55__)_ = 2.727, *P* = 0.029]. Subsequently, one-way repeated-measures ANOVA found no significant difference in pupil diameter index among six paradigms at fatigue level 1 [*F*_(__5,55__)_ = 1.796, *P* = 0.129] and fatigue level 4 [*F*_(__5,55__)_ = 0.170, *P* = 0.973].

One-way repeated-measures ANOVA was also used to analyze the difference in pupil diameter index for all six paradigms between fatigue level 1 and level 4 and found a significant decrease in paradigms A, B, C, D, and E [*F*_(__1,11__)_ = 18.291, *P* = 0.001 for paradigm A; *F*_(__1,11__)_ = 15.803, *P* = 0.002 for paradigm B; *F*_(__1,11__)_ = 15.226, *P* = 0.002 for paradigm C; *F*_(__1,11__)_ = 8.134, *P* = 0.016 for paradigm D; *F*_(__1,11__)_ = 13.177, *P* = 0.004 for paradigm E). However, there was no obvious change in pupil diameter index for paradigm F [*F*_(__1,11__)_ = 0.091, *P* = 0.769]. This also revealed that paradigm F had better anti-fatigue efficacy than the other five paradigms. Hence, the anti-fatigue performance of the six paradigms based on the pupil diameter index was as follows: F > E = A = B = C = D.

### Comparison of α + θ Index

The α + θ band corresponding to each paradigm was normalized for each subject by his/her respective baseline α + θ band of the first three pre-experimental trials of each paradigm. Figure 5B presents the normalized α + θ index between fatigue level 1 and fatigue level 4 for six stimulus paradigms over 12 subjects. Two-way repeated-measures ANOVA revealed that the interaction of two factors of “stimulus paradigm” and “fatigue level” was non-significant in normalized α + θ index [*F*_(__5,55__)_ = 1.930, *P* = 0.104]. The factor of “stimulus paradigm” had a significant effect on α + θ index [Greenhouse–Geisser *F*_(__2.466,27.128__)_ = 13.166, *P* < 0.001], and the corresponding Bonferroni *post-hoc* analysis of α + θ index among six paradigms is shown in Table 6. The factor of “fatigue level” also had a significant effect on α + θ index [*F*_(__1,11__)_ = 9.028, *P* = 0.012]. The α + θ index and its change of paradigms A, B, and C were similar, revealing that paradigms A, B, and C had a similar anti-fatigue performance. Similarly, paradigms D and E also had a close anti-fatigue performance. Although the α + θ index of paradigm F was slightly higher than those of other paradigms at fatigue level 1, there was little change in the α + θ index between fatigue level 1 and level 4, demonstrating that paradigm F had better anti-fatigue efficacy than the other five paradigms. Hence, the anti-fatigue performance of six paradigms based on the α + θ index was as follows: F > A = B = C = E ≥ D.

**TABLE 6:** Bonferroni *post-hoc* analysis of the α + θ index among six paradigms.

Paradigm	B	C	D	E	F
A	*P* = 1.000	*P* = 1.000	*P* = 0.362	*P* = 0.010	*P* = 1.000
B	–	*P* = 0.479	*P* = 0.072	*P* < 0.001***	*P* = 0.035*
C	–	–	*P* = 0.278	*P* < 0.001***	*P* = 0.110
D	–	–	–	*P* = 1.000	*P* = 1.000
E	–	–	–	–	*P* = 0.024*
F	–	–	–	–	–

***P < 0.001; **P < 0.01; *P < 0.05.

## Discussion

In this study, we used four indices of α + θ index, pupil diameter index, NASA-TLX, and SSVEP amplitude and SNR to compare the mental fatigue and anti-fatigue performance of six paradigms in SSVEP visual acuity assessment. First, as for α + θ index, the θ waves tend to appear during meditative, drowsy, hypnotic, or sleeping states, and the increase in θ waves is related to performance decrements on task (Klimesch, 1999; Xie et al., 2016). The α waves appear during wakeful relaxation with closed eyes at decreased attention levels and in a drowsy but wakeful state, and the increase in α waves associated with fatigue is related to the increased mental effort to maintain vigilance level (Cao et al., 2014; Kathner et al., 2014). More specifically, the decreased attention and arousal level caused by mental fatigue are associated with the global increase in the θ and the α activities. Hence, the α + θ index shows a significant increase associated with the increasing fatigue level and also related to the mental alertness level (Eoh et al., 2005; Cao et al., 2014). Second, as for the pupil diameter, it is also a well-documented psycho-physiological proxy of effort, load on memory, and arousal (Peysakhovich et al., 2015), and the decrease in pupil diameter is related to deep breathing, mental work, and sleep. Hence, the increase of mental fatigue coincides with the decrease in pupil diameter (Hopstaken et al., 2015b). Third, NASA-TLX is a gold standard for measuring subjective mental fatigue across a wide range of applications (Hart and Staveland, 1988). Finally, previous studies have proved that the SSVEP amplitude and SNR can be significantly affected by the increasing fatigue level, and the amplitude and SNR of the elicited SSVEP are easily affected by mental states, fatigue, and degree of attention level (Cao et al., 2014; Xie et al., 2016).

This study focused on the mental fatigue effects caused by the long-time SSVEP stimulus of six stimulus paradigms. The results of all the indices of α + θ index, pupil diameter index, NASA-TLX, and SSVEP amplitude and SNR showed that paradigm F of motion expansion–contraction had a superior anti-fatigue efficacy than the other five paradigms of conventional onset mode or pattern reversal SSVEP stimulation during prolonged SSVEP experiment. The paradigm D of brief-onset mode showed the lowest anti-fatigue efficacy, and the other paradigms A, B, C, and E of pattern reversal SSVEP stimulation paradigms showed a similar anti-fatigue efficacy, which was between paradigms D and F. These indices of mental fatigue showed a good agreement. The results showed the anti-fatigue performance calculated averagely through all the four indices of mental fatigue estimation of six paradigms as follows: F > E ≥ A = B = C > D. Besides that, the decrease of SSVEP amplitude and SNR caused by mental fatigue during prolonged EEG experiment especially in paradigms A, B, C, D, and E may consequently deteriorate the SSVEP visual acuity assessment since the threshold determination criterion of SSVEP acuity is related to the amplitude and SNR of SSVEP response (Zheng et al., 2019a). Hence, we recommended paradigm F of oscillating expansion–contraction concentric rings as the stimulation paradigm in SSVEP visual acuity.

The reason for paradigm F to have the highest anti-fatigue property may be because of its uniform brightness and position changes rather than luminance alternations when presented to the subject, which overcame the problem of visual fatigue caused by uncomfortable light twinkling and contrast changes in the pattern reversal and brief-onset mode (Xie et al., 2012, 2016; Han et al., 2018; Yan et al., 2018). According to the theory of visual pathways, the visual system is divided into two major pathways of the parvocellular pathway and the magnocellular pathway (Pokorny and Smith, 1997). The magnocellular pathway contains the detection of dynamic motion and depth, whereas the parvocellular pathway contains the detection of spatial contrast and color information, with a slower propagation than the magnocellular pathway. Previous studies have proposed that attention uses the faster and more dominant signals of the magnocellular pathway to give priority to stimuli and simultaneously enhance the activity of the parvocellular pathway (Pokorny and Smith, 1997; Di Russo and Spinelli, 1999; Yeshurun and Sabo, 2012). If attentional networks are more reliant on parvocellular pathways, extra reaction time and demand are required for attention (Li et al., 2007; Laycock et al., 2008). Hence, the attention demand may be alleviated in motion expansion–contraction mode in paradigm F, while the contrast change in paradigm D of brief-onset mode may be a bit intense, resulting in the increase of attention demand.

There were also some limitations in this study that should be weighted. First, although the temporal frequency of 7.5 Hz was often used in SSVEP visual acuity (Almoqbel et al., 2011; Hamilton et al., 2020), the only one temporal frequency did not fully consider all relevant research since mental fatigue was limited by stimulus frequency to some extent (Won et al., 2016). Second, the number of trials and the time spent on one run were not necessarily the same as those of the SSVEP acuity test in clinical experiments, which may also have a certain influence on the results. Third, we used binocular rather than monocular viewing in our study, and the two may be not completely equivalent. Fourth, we used a consistent spatial frequency rather than a set of sweep spatial frequencies similar to the SSVEP visual acuity experiment, which may also lead to some difference in results from the actual experiment. Finally, in this study, compared to pupil diameter index and α + θ index, there were no corresponding baseline of SSVEP amplitude and SNR in the first three pre-experimental trials of each paradigm because of no visual stimulus during the three trials, so SSVEP amplitude and SNR were not normalized by respective baseline.

## Conclusion

To conclude, this study has explored the anti-fatigue performance of six stimulus paradigms (reverse vertical sinusoidal gratings, reverse horizontal sinusoidal gratings, reverse vertical square-wave gratings, brief-onset vertical sinusoidal gratings, reversal checkerboards, and oscillating expansion–contraction concentric rings) used in SSVEP visual acuity assessment. Four indices of α + θ index, pupil diameter index, NASA-TLX, and SSVEP amplitude and SNR were proposed to estimate mental fatigue quantitatively. These indices of mental fatigue showed a good agreement. The results showed that the paradigm of motion expansion–contraction had a superior anti-fatigue efficacy than the other five paradigms of conventional onset mode or pattern reversal mode during prolonged SSVEP experiment. The paradigm of brief-onset mode showed the lowest anti-fatigue efficacy, and the other paradigms of pattern reversal mode showed a similar anti-fatigue efficacy, which was between motion expansion–contraction mode and onset mode.

Except for brief-onset vertical sinusoidal gratings, the four commonly used stimulus paradigms (i.e., reverse vertical sinusoidal gratings, reverse horizontal sinusoidal gratings, reverse vertical square-wave gratings, and reversal checkerboards) in SSVEP acuity assessment had a relatively good anti-fatigue property, indicating that mental fatigue could not affect the SSVEP acuity estimation too much when using the four stimulus paradigms. As for the paradigm of oscillating expansion–contraction concentric rings, it had the highest anti-fatigue property, and we recommended the oscillating expansion–contraction concentric rings as the stimulus paradigm in SSVEP acuity assessment.

## Data Availability Statement

The raw data supporting the conclusions of this article will be made available by the authors, without undue reservation, to any qualified researcher.

## Ethics Statement

The studies involving human participants were reviewed and approved by the institutional review board of the Xi’an Jiaotong University. The patients/participants provided their written informed consent to participate in this study. Written informed consent was obtained from the individual(s) for the publication of any potentially identifiable images or data included in this article.

## Author Contributions

XZ contributed to the study design, data acquisition, analysis, interpretation, manuscript writing, and revision. GX contributed to the study design and the approval of the final version for publication. YZ contributed to the statistical analysis and manuscript drafting. RL and KZ contributed to the data analysis and interpretation. YD contributed to the manuscript writing and revision. JX provided the experimental equipment and approved the final version for publication. SZ conceptualized the study. All authors contributed to the article and approved the submitted version.

## Conflict of Interest

The authors declare that the research was conducted in the absence of any commercial or financial relationships that could be construed as a potential conflict of interest.
